# Classification of non-coding variants with high pathogenic impact

**DOI:** 10.1371/journal.pgen.1010191

**Published:** 2022-04-29

**Authors:** Lambert Moyon, Camille Berthelot, Alexandra Louis, Nga Thi Thuy Nguyen, Hugues Roest Crollius

**Affiliations:** Ecole Normale Supérieure, PSL Research University, CNRS, Inserm, Institut de Biologie de l’Ecole Normale Supérieure (IBENS), Paris, France; University College London, UNITED KINGDOM

## Abstract

Whole genome sequencing is increasingly used to diagnose medical conditions of genetic origin. While both coding and non-coding DNA variants contribute to a wide range of diseases, most patients who receive a WGS-based diagnosis today harbour a protein-coding mutation. Functional interpretation and prioritization of non-coding variants represents a persistent challenge, and disease-causing non-coding variants remain largely unidentified. Depending on the disease, WGS fails to identify a candidate variant in 20–80% of patients, severely limiting the usefulness of sequencing for personalised medicine. Here we present FINSURF, a machine-learning approach to predict the functional impact of non-coding variants in regulatory regions. FINSURF outperforms state-of-the-art methods, owing in particular to optimized control variants selection during training. In addition to ranking candidate variants, FINSURF breaks down the score for each variant into contributions from individual annotations, facilitating the evaluation of their functional relevance. We applied FINSURF to a diverse set of 30 diseases with described causative non-coding mutations, and correctly identified the disease-causative non-coding variant within the ten top hits in 22 cases. FINSURF is implemented as an online server to as well as custom browser tracks, and provides a quick and efficient solution to prioritize candidate non-coding variants in realistic clinical settings.

## Author summary

Genetic diseases are caused by DNA mutations disrupting gene sequences, but also non-coding regions that regulate their expression. Identifying such non-coding mutations is difficult, because the precise location and function of regulatory regions remain poorly characterized. When analysing complete genome sequences from patients, clinicians must rely on bioinformatic tools to rank the most likely candidate mutations. Here we present FINSURF, a new machine-learning method trained to recognise non-coding mutations likely to cause disease. FINSURF outperforms state-of-the-art methods by considering a composite benchmark of control mutations with no described phenotypic effect. A novel feature in FINSURF also consists in associating non-coding mutations to target genes whose expression might be affected, enabling users to narrow down on genes relevant to the disrupted function or disease, if known. When applied to complete human genomes containing millions of benign mutations, in 73% of cases FINSURF correctly identified mutations causing varied genetic diseases in the top 10 scores. FINSURF is available for download, as a genome score track and as an online server.

## Introduction

Whole genome sequencing (WGS) is increasingly used to diagnose pathogenic genetic variants in patients. However, interpretation of whole genome sequencing data remains virtually restricted to the ~2% that encode proteins, because the only mutations we functionally understand well are those affecting codons and splice sites. Disease-causing non-coding variants, on the other hand, presumably operate by deregulating gene expression. Regulatory mutations causing genetic diseases are known to show a wide range of penetrance in mammals [1,2]. Still, numerous examples demonstrate that a single base pair change in a transcription factor binding site or in a gene promoter can disrupt gene expression and cause a pathology [2–6]. Identifying such regulatory variants remains eminently challenging, as it requires demonstrating that the mutation modifies gene expression timing, intensity or cell type, and leads to a disease phenotype. This may explain why large fractions of patients participating in WGS cohorts do not receive a molecular diagnosis [7,8].

A first issue to identify candidate pathogenic variants in patients is the sheer volume of genetic variants to consider. A WGS dataset delivers 4 to 5 million variants per individual. Less than 10% are present at high frequency in the human population (more than 5% of individuals), the vast majority (>90%) are present at lower frequencies [8], and a few thousand are unique to each genome [9]. Ultra-rare variants, among which a mutation causing a highly penetrant genetic disease might be sought in priority, therefore amount to tens of thousands of candidates. Secondly, the catalogue of regulatory elements in the human genome is still incomplete. Tremendous progress has been made in characterising the properties of noncoding regions using genome-wide assays in many cell types, including the ENCODE [10] and the Roadmap Epigenomics project [11]. However the relationship between epigenomic signals as well as their amount of true and false positives have yet to be established [12]. Studies consistently report more than two million predicted regulatory regions in the human genome [11,13], but most of these are not experimentally validated. Large-scale reporter assays can quantify the impact of individual variants on gene expression, but being ectopic, they do not account for the complex cognate genomic context of true regulatory regions [14,15]. Finally, linking a regulatory mutation to the gene whose expression is modified remains a critical step to demonstrate disease causality. Regulatory regions lie in vast expanses of non-coding DNA, sometimes hundreds of kilobases from their target gene. Different approaches have attempted to link regulatory elements to genes genome-wide, using correlated expression (e.g. the FANTOM project [16]), correlated chromatin states [13,17], physical contacts with a TSS (Capture-HiC [18,19]) or evolutionary linkage (e.g. PEGASUS [20]), but the specificity of these methods is unknown.

Considering the tremendous amount, diversity and complexity of information available on chromatin states, evolutionary conservation and genome topology associated with gene regulation, a number of computational methods based on machine learning have recently been developed to identify putatively functional non-coding variants [21] (Table C in S1 Text). Their aim is to integrate the data into a single statistical framework and rank variants through a score that reflects their functional importance or regulatory potential. Current methods suffer from three main limitations. First, specificity and sensitivity are typically evaluated using data similar to the data used for model training, and it is unclear how models generalise to new variants. Second, scores are over-simplified numerical values that do not capture the rich and heterogeneous set of annotations contributing to variant selection. Third, most methods do not assign candidate regulatory variants to a predicted target gene, or resort to a naive “nearest gene” approach to do so, even though this is incomplete in many cases [16,22,23].

Here we describe FINSURF, a method for ranking non-coding variants in the context of human diseases. FINSURF computes a functional score predictive of the damaging nature of the mutation, but also breaks the score down into quantified contributions from all the features, making it biologically interpretable. In addition, FINSURF associates variants to one or several putatively deregulated genes when possible, which can be confronted to known genes implicated in a disease. Using a realistic setup that replicates real patient WGS in a broad range of diseases, we demonstrate that FINSURF is able to pick out the correct damaging regulatory mutations from several million variants with high accuracy and precision.

## Material and methods

### Training datasets

As positive controls, we selected all variants labelled as Damaging Mutations from the Human Gene Mutation Database [5] (version Pro 2017.2). To focus on non-coding variants, we excluded variants that were annotated as protein-impacting and/or located within CDS according to the HGMD gene annotations. We additionally used the GENCODE [24] genome annotation (version 29 lift-over hg19) to further exclude variants located within CDS, start codon, stop codon, or splice sites. This lead to the identification of 880 non-coding damaging mutations. This entire set was included for the “Random” model, but reduced to 878 for the “Adjusted” model and to 877 in the “Local” model, as some variants did not have any appropriate negative controls (see hereafter for the description of these models and sampling schemes). Negative controls were sampled from a set of 38,056,330 variants for which no medical impact was found (accessed on 2017/09/05 from http://ftp.ncbi.nlm.nih.gov/pub/clinvar/vcf_GRCh37/archive_1.0/2017/).

A first set of 880,000 negative controls was sampled randomly after removing coding variants and indels, as no indels were present in the positive set (here-after named "Random"; 880 variants sampled per tree, over 1000 trees). This corresponds to a naive approach under which no particular bias is expected between the positive and negative controls aside from the differences in distributions of annotations. A second set of 6,113 negative controls was selected within 1,000 bp of positive control variants (here-after named “Local”). This control set was built to test separation of positive and negative controls at high genetic resolution. The third set (called “Adjusted”, which was used for the analysis) corresponds to a intermediate situation, where negative controls are sampled from cytogenetic bands containing at least one positive control to avoid artificially biasing negative controls towards non-functional genomic regions [25]. Additionally, proportions of negative controls in the different GENCODE biotypes were matched to those of positive controls. This correction aims at forcing the classification model to focus on the functional differences between positive and negative controls, rather than capturing differences that arise from a location bias (as positive controls are biased towards gene-proximal non-coding regions). In total, 67,089 negative controls were retained in this set.

### Annotation of non-coding variants

See Table A in S1 Text for a complete list of annotations. To build the classification model, we identified four groups of annotations to characterize non-coding variants. The first group corresponds to annotations related to sequence evolutionary conservation. PhyloP and PhastCons scores for the human genome (hg19 version) based on the 100 vertebrate multiple genome alignment were downloaded from the UCSC web browser. The same scores were obtained for the 20 primate genomes alignments for the hg38 version, and converted to hg19 coordinates using liftOver [26]. In addition, GERP scores and GERP elements were downloaded from the Sidow lab webpage (hg19 version). Finally, the Context-dependent tolerance score (CDTS) evaluates constraint at the human population level. Scores were obtained from the original publication [27] for hg19. The second group corresponds to annotations describing biochemical properties of the genome. The Roadmap Epigenomics project provides with 18 chromatin states inferred from combinations of histone modifications, across 98 cell types. This large amount of features would correspond to a very sparse set of annotations, a variant being associated to a single of the 18 chromatin states for a given cell type. As sparse annotations are poorly exploited by random forests, we aggregated these chromatin states per genomic position and counted the number of cell types corresponding to each chromatin state. We also selected three key histone marks of regulatory regions, H3K4me1, H3K4me3, and H3K27ac, and calculated the median Fold Change value at each genomic position across the same 98 cell types. Additionally, we downloaded two datasets related to transcription factor binding sites from the UCSC Genome Browser: TFBS identified as conserved between mouse, rat, and human; and clusters of TFBS identified in ChIP-seq peaks from 161 experiments across 91 cell types from the ENCODE project. A complementary dataset of TFBS from JASPAR, identified within ChIP-seq peaks, was obtained from Ensembl [28]. Finally, a dataset of DNaseI hypersensitive regions was obtained from the UCSC, corresponding to clusters identified in 125 cell types from ENCODE. A third group of features describe sequence properties related to variant locations. Three features were retained: CpG dinucleotides and CpG islands, downloaded from the UCSC, and variant type (transition, transversion, or INDEL). The last group gathers annotations from recently published datasets predicting regulatory regions in the genome together with their gene targets. We extracted predicted regulatory regions from the following datasets: GeneHancer [29] (version 4.4, accessed 2018-12-17), PEGASUS [20] (2018-12-17), FANTOM5 co-expressed regions [16] (2018-11-19), FOCS FANTOM5, FOCS ROADMAP DHS, and FOCS Gro-seq co-expression [17] (2018-12-11). We also included promoters (2kb upstream of transcription start sites) and UTR regions from all coding and non-coding genes from the GENCODE [24] dataset as potential non-coding regions of interest (v29lifthg19). These datasets were integrated by merging overlapping elements into regions with one or multiple evidences of predicted associations. This integration was not performed for GeneHancer, which was handled separately, as it does not provide a single score for each regulatory region/predicted target pair. A list of predicted targets was derived for each regulatory region by merging all predicted target genes across methodologies. In addition to these four groups of annotations, which are used for classification by the FINSURF model, other annotations were included for filtering and characterizing variants. Notably, we used the GENCODE (v29liftHg19) annotations for the locations of biotypes such as CDS, introns, etc. A total of 471,099,210 genomic positions were thus annotated with the set of descriptors, and evaluated for functional potential with FINSURF. For each position, 2 predictions are reported: one for transitions and one for transversions. Tabix-indexed tabular files for all chromosomes were generated, allowing the fast interrogation of these files for millions of variants of interest. Functional profiles are reported for all variants lying within annotated regulatory regions. For indels, all positions affected by the variant (as well as the one preceding and following positions) are evaluated, but only the highest score is reported.

### Model training and performance evaluation

We trained three Random Forest models (Random, Adjusted and Local, using different negative control sets) using the Scikit-Learn Python library v.0.20.2, with the following parameters: 1,000 trees, maximum depth = 15 nodes, minimum number of samples per leaf = 1. These parameters were optimized using a nested-cross validation evaluation of 400 models, exploring different range of values. Class size imbalance issues in the training set were solved by sampling with replacement a set of n random variants for each class, where n is the size of the smallest class (in this case, HGMD-DM non-coding variants; n = 880). Each tree in the Random Forest was then built from a different, balanced set of positive and negative controls. Model performance was evaluated using 10-fold cross-validation. We note that genetic variants in the training set are not necessarily independent, as they can be located at close genomic distance and thus share some of their features. This can lead to performance over-estimation when closely located variants of the same class are split into training and validation sets, which can artificially favour correct classification of the validation variants. To mitigate this problem, for cross-validation variants were separated by location, based on cytogenetic bands (Fig B in S1 Text). Model discrimination between variant classes was evaluated based on the Receiver-Operating Curve (ROC; true positive rate as a function of false positive rate) and the Precision-Recall Curve (PRC; proportion of true positives among all positives, as a function of the true positive rate). This second curve is of particular interest in the context of imbalanced learning, as it better captures how the proportion of true positives against false positives changes with increasingly lenient thresholds on the prediction score. For the Adjusted model, we maximized the F1-score, defined as the harmonic mean of the precision and recall, and obtained an optimal prediction score threshold of 0.51. This threshold was used to calculate the confusion matrix. Eight other methods were also evaluated using 10-fold cross-validations on the control dataset. Variants missing a score for any of the methods were excluded from the evaluation (average drop-out rate = 53%). Indels were scored as with FINSURF: all bases covered by the indel were scored, but only the highest scoring position was retained.

Finally, we applied each of the three FINSURF models (Random, Adjusted, Local) on the training datasets of the two other models, in order to evaluate the performance of each model across different genomic contexts. We re-used the 10-fold cross-validation scheme used for model training, but evaluated the 10 partial models using negative test sets sampled with either of the other two models. In order to make the performance curves comparable, an additional random sub-sampling was performed on the negative controls, so that the proportion of positives in the test-subset of each k-fold was as close as the one from the “Local” selection scheme (12.5%).

### Independent evaluation

We downloaded 448 disease-causing non-coding variants used to train the ReMM-Genomiser model [25]. Of these, we excluded 11 variants found within coding regions according to GENCODE v29. We then intersected these variants with our training set of regulatory HGMD-DM variants and excluded overlaps. This resulted in a set of 92 non-coding regulatory variants independent from our training positive controls, which were used to compute ROC and PRC curves. Of these, 30 were dropped during the comparison against other methods, as some models did not provide scores for these positions, leaving 62 variants for the comparison. Negative controls (N = 17,122) were sampled from the ClinVar dataset, using the Adjusted sampling protocol. For analysis of a realistic genome-wide VCF, we further excluded disease-causing non-coding variants located within 1,000 bp of an HGMD-DM variant from the FINSURF training dataset, and specifically focused on 49 variants that represent a set of fully independent regulatory variants with respect to our model. All non-coding variants (according to GENCODE v29) from the Illumina Platinum genome NA12877 [30] were annotated and scored with FINSURF as a realistic genetic background.

### Feature contributions and clustering

Feature contributions were calculated using a more computationally efficient, in-house re-implementation of the treeinterpreter package (https://github.com/andosa/treeinterpreter; https://arxiv.org/abs/1906.10845), which calculates the average decrease in Gini impurity index across all trees for each feature/variant combination. To avoid overfitting, feature contributions for a particular variant were calculated only from trees where this variant was not used for training. K-means clustering was performed on the feature contributions vectors for the positive control variants to explore structure in the training set. Different K values from 2 to 19 were explored, and the optimal K was selected using maximization of the silhouette score and inertia minimization with Scikit-Learn v.0.20.2. For each cluster, mean feature contributions were calculated to obtain the average functional profile. To translate these feature contributions into feature values, we calculated the feature effect size for variants within a cluster against variants from other clusters combined with negative controls. This comparison highlights distinctions between positive and negative controls that are specific to this cluster. Effect size was calculated depending on feature distribution: Cohen’s h for binary features, and Cohen’s d for discrete and continuous features:

$$d_{cohen}=\frac{\left({\bar{x}}_{A}-{\bar{x}}_{B}\right)}{{std}_{pooled}}$$

$$h_{cohen}=2\left(arcsin(\sqrt{\left(p_{A}\right)}-\sqrt{\left(p_{B}\right)})\right)$$

### Disease association analysis

We collected sets of genes from OMIM [31], associated with 30 diseases caused by 49 fully-independent non-coding variants from the ReMM-Genomiser [25] training dataset (see ‘Independent evaluation’ above). Each disease was associated with a single gene, except for “Cerebral Amyloid Angiopathy, APP-related” (OMIM:605714) for which 3 genes were retrieved from the OMIM webpage. Variants within regulatory regions with predicted targets that contained the identified disease-gene were selected and ranked based on their FINSURF score.

## Results

### FINSURF accurately distinguishes true regulatory variants from a variety of negative controls

**Fig 1:**
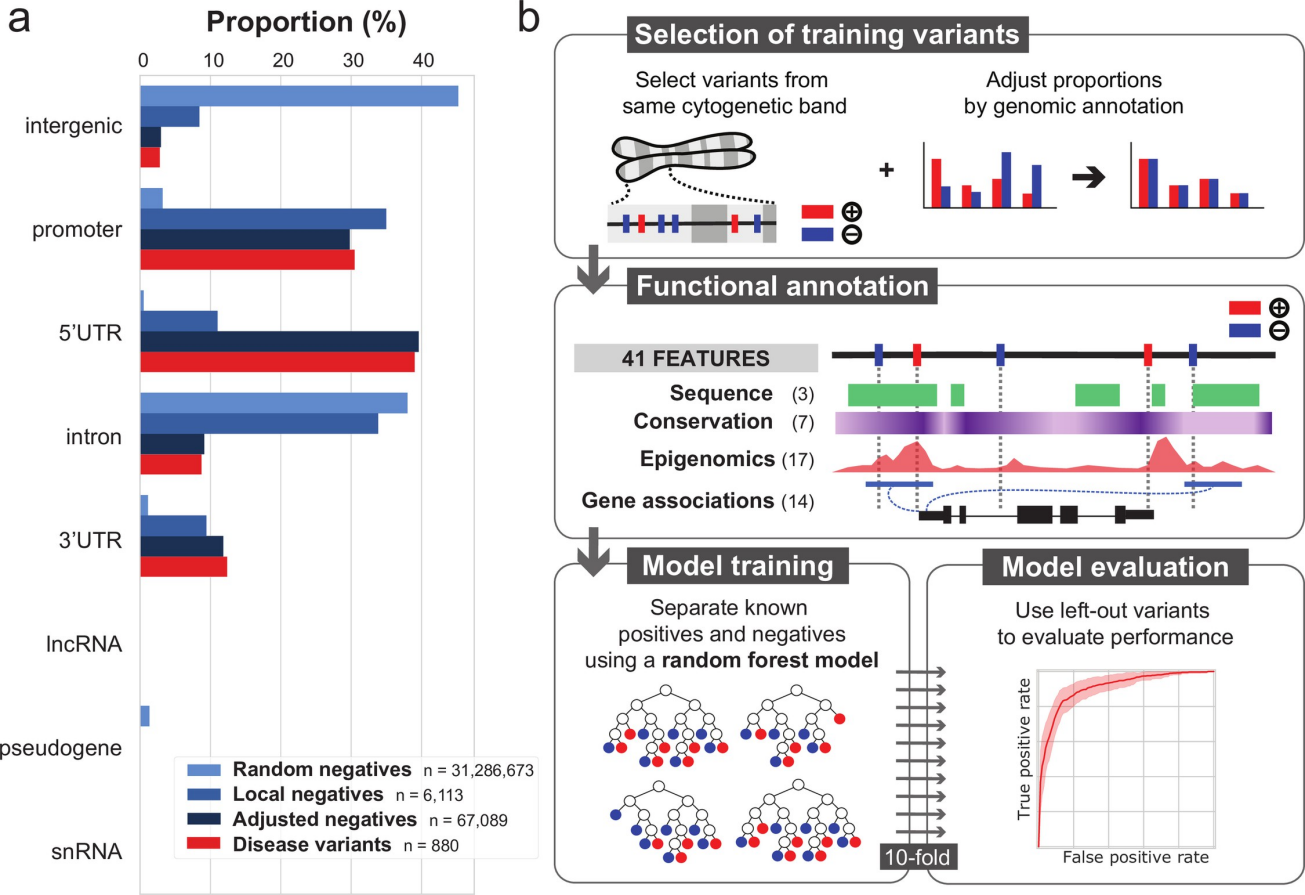
FINSURF design strategy. **a**. Percentage of genetic variants intersecting GENCODE biotypes across benign variants (shades of blue, corresponding to different sampling strategies) and damaging variants from the HGMD database (red). **b**. The final pipeline leading to the FINSURF model. Control negative variants were sampled using the Adjusted strategy. Both the negative and positive sets were annotated with 41 features, and a random forest classifier was trained to distinguish them on this basis. Ten iterations were performed, each time using 9/10 of the data, while testing performances on the remaining 1/10 which had not been used for training.

We trained random forest classifiers [32] using 880 experimentally validated, non-coding regulatory variants as a positive training set, identified in the HGMD [5] database as Damaging Mutations (hereafter named “HGMD-DM” variants). We sampled control variants from 31 million non-coding variants with no clinical significance in the ClinVar [33] database as negative training sets. Importantly, we observed that negative and positive variants are unequally distributed with regard to genomic features such as introns, intergenes or promoters (Fig 1A). This probably reflects a mixture of underlying biology of regulatory variants and bias in the HGMD database. We therefore defined three negative training sets to explore the impact of genomic variant distribution on the model ability to discriminate non-coding regulatory variants. Briefly, two extreme models were built: one with randomly sampled negative controls (“Random”), and one with negative controls sampled within 1 kb of variants from the positive set (“Local”; Material and Methods). The first model learns from large numbers of variants across the entire genome; however, the model may be overly naive, as genomic distribution biases in the training set may result in poor discrimination between closely located variants. The second model separates positive and negative variants at high resolution; but it explores a small fraction of the genome and may not generalize. We also defined an intermediate model (“Adjusted”), where we sampled negative controls from cytogenetic bands containing positive controls, a similar strategy to ReMM-Genomiser [25], and then sub-sampled to match the proportions of positive and negative variants within genomic feature intervals as defined by GENCODE biotypes (Material and Methods, Fig 1A and 1B). During model training, we ensured that contributions from positive and negative training sets remain balanced (Material and Methods). We assembled a compendium of 41 genomic features to deeply annotate all variants, including evolutionary sequence conservation [27,34–37], biochemical composite annotations from hundreds of biological contexts [11,13,38], sequence features, and predicted regulatory element–gene interactions [16,17,20,29] (Table A in S1 Text and Fig 1B).

We evaluated the model’s abilities to distinguish regulatory from non-regulatory variants by performing 10-fold cross-validation. The positive and negative variant sets were each divided in ten subsets, and the random forest model was trained on 9 parts and its performances evaluated on the 10^th^, which contains variants not used for the training. This process was run 10 times, using each subset as the left-out evaluation subset once (Fig 1B).

**Fig 2:**
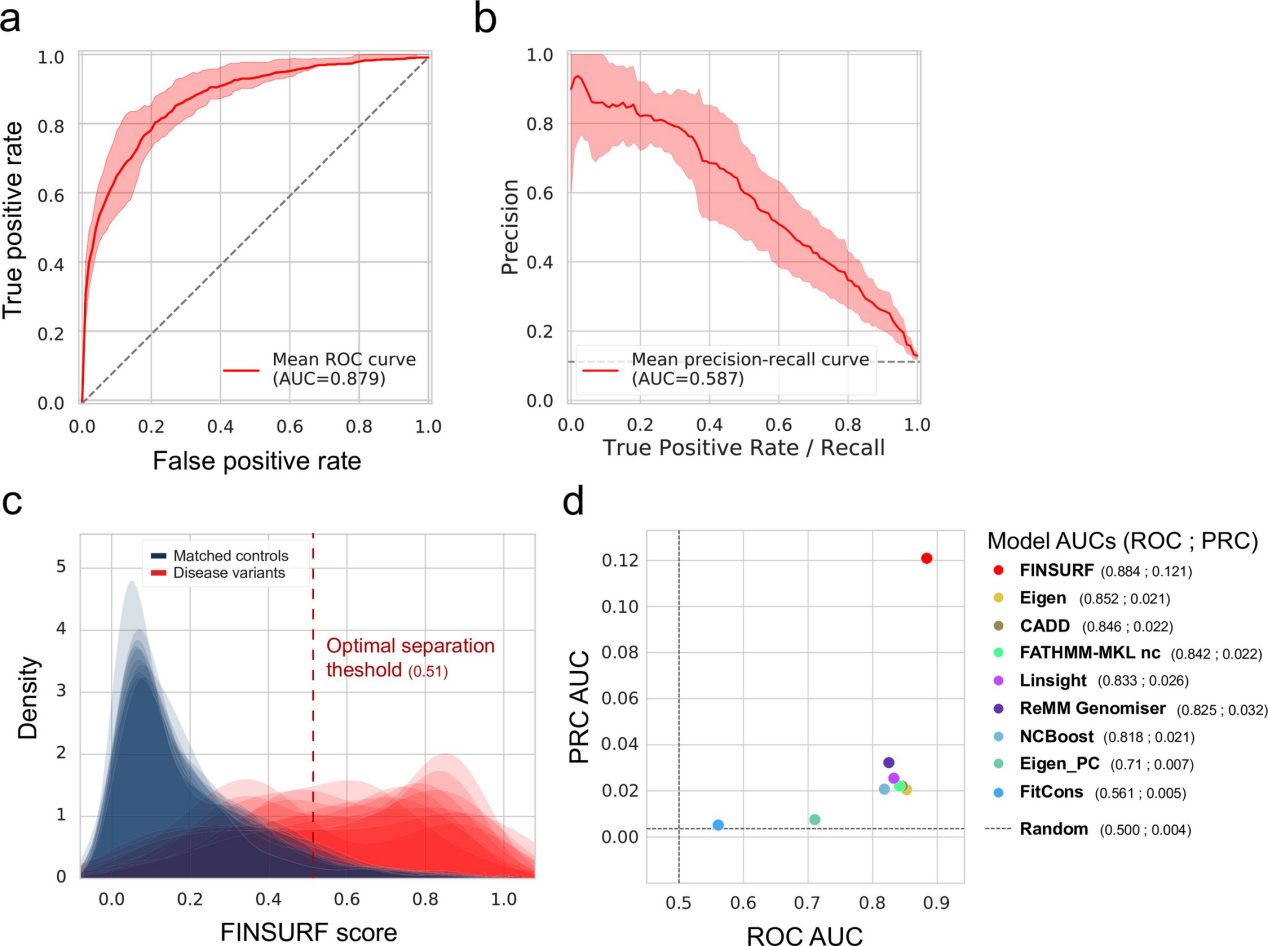
FINSURF performances. **a**. Receiving Operating Curve (ROC) after a 10-fold training procedure. The average curve is shown in bold red and the 95% confidence interval is indicated by a pink shading, with the mean Area Under Curve (AUC) reported in the bottom right. The dashed diagonal line indicates the distinction between positives and negatives expected by chance (AUC = 0.50). **b.** Precision Recall Curve (PRC) computed from the same 10-fold training procedure. As for the ROC, the average curve is shown in bold red and the 95% interval is indicated by a pink shading, with the mean Area Under Curve (AUC) reported at the bottom. The dashed diagonal line indicates the amount of true positive to be recovered by a model predicting all variants as positive, fixed to 12.5%. **c**. Distributions of FINSURF scores in the test set for each of the 10-fold trainings. Scores for negative variants are shown in blue, and for positive variants in red. The vertical dashed line represents the optimal score threshold (0.51) to separate positives from negatives (Material and Methods). **d**. ROC curves comparisons between FINSURF and eight other methods on a set of 62 variant independent from the training set of FINSURF. AUC values for each method are indicated in the legend.

The Random model performs best, with an area under the curve (AUC) of the Receiving Operator Curve (ROC) of 0.957 and AUC of the Precision-Recall Curve (PRC) of 0.823 (Fig A in S1 Text). Following are the Adjusted model and the Local model, in order. This performance gradient is consistent with the nature of the negative training sets. Indeed, random non-coding variants largely fall in repetitive and non-functional genomic DNA, and are easily distinguished from regulatory regions, but discrimination becomes harder as the negative set becomes more similar to the positive set. While impressive performances can be achieved by using highly contrasted negative and positive sets, such a model may perform poorly at separating the wheat from the chaff amongst closely located variants. To test this, we applied each of the three trained models to discriminate the positive variant set from the negative sets of the other two models (Fig A in S1 Text). As expected, all three models display lower performance when discriminating variants distributed differently from their own training set. Nonetheless, the Adjusted model generalises well: it performs similarly with randomly sampled negatives and its own adjusted set of negatives (ROC AUCs = 0.948 and 0.879, respectively), and just slightly underperforms compared to the Local model when using closely located positive and negative variants (ROC AUCs = 0.841 and 0.796, respectively). Because of its high performances and its ability to generalize genome-wide as well as to discriminate locally, Adjusted represents an advantageous model on which we base the rest of this study (Fig 2A and 2B). We named this new model FINSURF, for Functional Interpretation of Non-coding Sequences Using Random Forests. For each variant, FINSURF provides a score corresponding to the proportion of decision trees in the random forest classifying this variant as regulatory, as well as a description of genomic features that contributed to this classification, which we refer to as a “functional profile”. As the functional impact of non-coding indels is heterogeneous and not well characterised, FINSURF will report the highest scoring position covered by the indel, including the two flanking positions, as a general solution. In addition to its score, each variant is associated with a list of putative target genes based on the union of publicly available resources linking putative regulatory sequences to target genes, including biochemical co-activation and conserved physical linkage (PEGASUS, GeneHancer, FANTOM5, FOCS, GENCODE promoters and UTRs; see Material and Methods: Annotation of non-coding variants and Table B in S1 Text). The FINSURF score can be used to annotate, classify and rank variants relative to each other, while genomic features and target genes can be used to further refine candidate pathogenic variant searches.

### Evaluation against alternative methods and variants

We next evaluated FINSURF against eight existing methods designed to assess the functional impact of non-coding variants. For this, we reused the same 10-fold cross-validation variant subsets used to evaluate FINSURF, with negative variants sampled from the Adjusted set. We scored each validation subset with the other methods as well, and we compared respective performances using ROC and PRC AUC (panel a of Fig C in S1 Text). FINSURF outperforms all methods according to the ROC AUC values (0.819), and is second best according to the PRC AUC values (0.486) after ReMM-Genomiser [25] (PRC AUC of 0.512). Of note, ReMM-Genomiser, NCBoost [39] and FATHMM-MKL are all trained on the HGMD-DM positive variants. These methods are therefore placed in overly favourable conditions, as variants used for their training likely appear in our validation subsets, while FINSURF is entirely evaluated on left-out variants. These results confirm that FINSURF is highly efficient to identify disease-causing, penetrant non-coding genetic variants of the type found in the HGMD-DM resource.

To verify that FINSURF’s performances extend to an independent set of non-coding regulatory mutations, we collected non-coding variants used for training ReMM-Genomiser but absent from the HGMD-DM resource. This small but independent dataset comprises 92 mutations, of which 62 can be scored by all eight methods, including 41 SNV and 21 INDELS. For the negative set, 31,564 variants (of which 17,122 can be scored by all methods) were selected from the non-coding and clinically non-significative ClinVar set, following the Adjusted sampling scheme and excluding those already used to train FINSURF. Remarkably, FINSURF again outperforms all alternative methods (Fig 2D and panel b of Fig C in S1 Text) despite some methods having been trained using this set of positive variants. Taken together, these results demonstrate that the combination of a carefully designed set of negative variants, a deep annotation and the optimization of a random forest classifier lead to advanced abilities to identify functional non-coding variants.

### FINSURF scores can be decomposed in biologically interpretable measurements

While accuracy is critical, most advanced statistical classification methods, including random forests, result in numerical scores, weights or probabilities that cannot be directly interpreted biologically. In addition to the score, FINSURF provides an array of descriptors for every biological feature and every variant, which serve to interpret how the model reached its conclusions. First, the Feature Importance measures how much a given biological feature contributed to discriminating positive and negative variants during training, averaged across all nodes and decision trees where the feature was sampled (Material and Methods) [40]. Feature Importance thus provides a feature-centric view of their relative discriminatory power. Second, Feature Contributions are variant-centric measures describing how each feature individually contributed in classifying a particular variant as positive or negative [41].

In agreement with previous models [25,39,42], the relative Feature Importances from the FINSURF model highlight the major influence of evolutionary sequence conservation scores in classifying regulatory variants (Fig D in S1 Text), with the GERP [37] score computed on a multiple alignment of 34 mammalian genomes largely dominating all other features. Additionally, the maximum predicted motif score within clusters of overlapping transcription factor binding sites (TFBS; Clustered TFBS max score) also plays a major role. Other prominent features include promoter segments and H3K27ac signals [11], as well as enhancers from the GeneHancer [29] collection. Together, these results confirm that FINSURF exploits diverse features consistent with different regulatory functions to identify non-coding pathogenic mutations.

**Fig 3:**
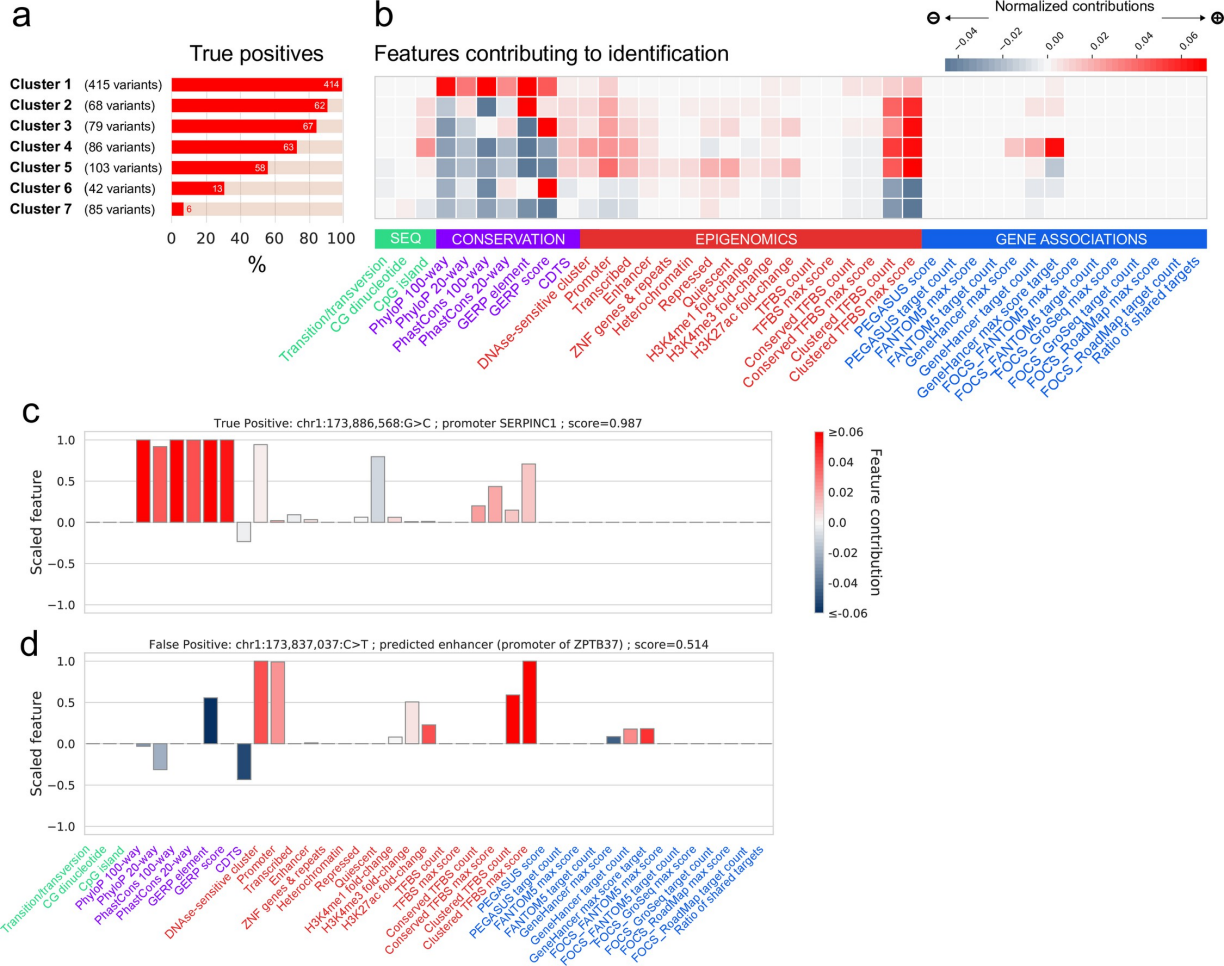
Feature contributions. **a**. The 880 positive variants were clustered using K-means into 7 clusters based on the contributions of all 41 features to their FINSURF score. Variants were classified as true positives or false negatives using the optimal score threshold (0.51). **b**. Average feature contributions in each cluster. The grey-red gradient reflects the normalized contribution of each feature and is relative across the entire grid. Features are grouped by functionally relevant categories (denoted by green, purple, red and blue colours). **c.** Functional profile of a True Positive variant, characterized as a disease-causing mutation impacting the SERPINC1 promoter. The heights of bars represent each of the features, rescaled between -1 and 1 from their distribution over the 400Mb of regulatory regions. The colours represents the feature contributions, highlighting which feature contributed positively (red) or negatively (blue) to the prediction score. **d.** Functional profile of a False Positive variant, passing the optimal threshold of 0.51, and found in regulatory regions also associated to SERPINC1.

Feature Contributions in turn allow users to investigate the biological properties of individual or groups of variants, and how they contributed to their classification. As an example, we relied on these variant-specific vectors to group the 880 HGMD-DM positive variants into seven clusters (Fig 3A and 3B, Fig E in S1 Text), revealing heterogeneity in the positive training set. First, the largest cluster by far (415 variants) contains 99.8% true positives after classification by FINSURF, and is characterised by a strong evolutionary conservation signal. Second, remaining clusters with more than 50% true positives rely less consistently on sequence conservation but are all characterised by a substantial overlap with predicted TFBS clusters. Finally, clusters 6 and 7 are two small clusters where FINSURF displays low accuracy (42 and 85 variants; false negative rate > 60%). Variants in these clusters display characteristics typical of variants from the negative set and have nearly no features contributing positively to their classification. This observation could be caused by insufficient coverage of their regulatory features in the collection used by FINSURF, possibly because they are condition-dependent, or that despite manual curation of the HGMD database, these variants are in fact not regulatory.

FINSURF additionally provides a graphical display tool to investigate the functional properties of specific variants. To illustrate this functionality, we explored the functional profiles of two example variants located in the vicinity of *SERPINC1*, one correctly classified as a regulatory variant (Fig 3C) and one non-functional but misclassified (Fig 3D). The first example case was identified by FINSURF as functional based on its strong evolutionary conservation, as highlighted by the Feature Contribution values. This variant also overlaps a promoter region, a TFBS cluster, and is quiescent in many tissues, but these features only weakly contributed to the classification. The second variant shows no evidence of medical relevance in the ClinVar database, but FINSURF mis-identified it as functional. The functional profile reveals that this variant is located within a promoter, overlaps a TFBS cluster and corresponds to an open chromatin region in many tissues as revealed by DNAse sensitivity, which jointly contributed to its misclassification.

### From whole genome sequences to pathogenic mutations

**Fig 4:**
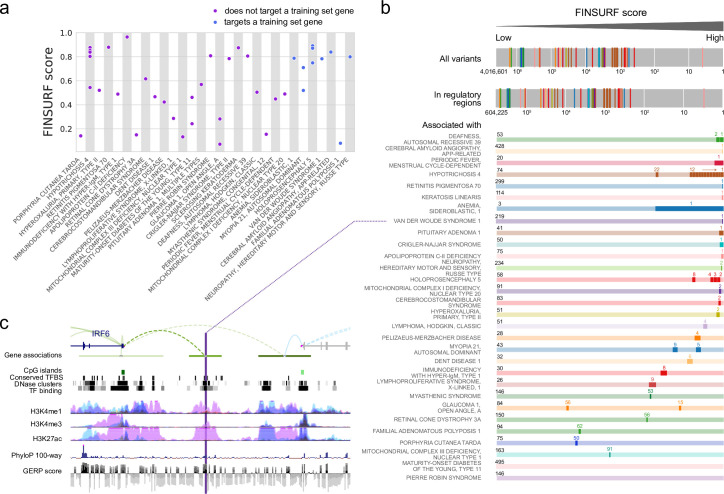
Application to medical genetics. **a**. A set of 49 regulatory variants causing human diseases (x-axis) not used for training were scored by FINSURF (y-axis). Eleven variants target a disease gene that is also targeted by a training variant (in blue), while 38 variants are totally independent (in purple). **b**. The 49 variants were seeded amongst over 4 million variants from a representative, otherwise healthy individual human genome, and their respective ranks are shown in the top bar (log scale; colors represent different diseases). When pathogenic and background variants are restricted to putatively functional non-coding sequences based on molecular or evolutionary evidence, ranking remains uninformative (second bar). However, when filtering for variants associated with disease genes, disease-causing mutations generally show high-ranking positions (coloured bars; total number of non-coding variants associated each disease indicated on the left; pathogenic variants highlighted in dark, with their rank above). **c**. Detailed genomic context for a non-coding mutation causing van der Woude syndrome 1 (MIM 119300), which is located in an enhancer ~30 kb in 5’ to the TSS of its target gene, interferon regulatory factor 6 (IRF6). Gene associations are from the GeneHancer collection, and depict the enhancer (green horizontal bar) with the link to its predicted target gene (dashed arc). All tracks are from the UCSC genome browser.

Given its high discrimination power and ability to generalize well across the genome, FINSURF is theoretically well suited to assist identification of pathogenic mutations when analysing Whole Genome Sequences (WGS) from patients. To test this, we generated realistic synthetic genomes that replicate a typical clinical situation, where a patient’s genome is sequenced to diagnose the molecular cause of a known disease, but no coding mutation can be detected in any associated gene and a regulatory mutation is suspected. We focused on the set of 92 curated non-coding variants used by REMM-Genomiser for training, known to cause 56 different genetic diseases, and that were not used to train FINSURF. Despite their independence from the HGMD-DM training set, 43 variants still lied in the immediate vicinity of mutations used for training and likely share some of their biological annotations, which translated in higher but possibly overfit FINSURF scores (panel a of Fig F in S1 Text). To alleviate this bias, we removed all variants located within 1,000 bp from any variant that FINSURF used during training, leaving 49 variants causing 30 diseases. These 49 variants display a wide distribution of FINSURF scores ranging from 0.072 to 0.965 (Fig 4A).

Then, we seeded these pathogenic variants amongst 4,016,599 genetic variants identified in a reference donor from the Illumina Platinum Genome^23^ collection, and scored the resulting synthetic genome with FINSURF. Unsurprisingly, the 49 pathogenic variants rank higher than average, with the majority (82%) comprised in the top 5% (Mann-Whitney test, p value = 1.23 10^−29^, Fig 4B). However this enrichment is of little practical use, as only one pathogenic variant scores in the top 100 variants which could realistically be investigated further by molecular biology techniques. This is an underappreciated limitation of pathogenic variant identification methods applied to real WGS data, where causative variants are vastly outnumbered by other variants, some of them with regulatory functions.

We show next how the regulatory and gene target predictions built into FINSURF dramatically increase its accuracy picking out disease-relevant regulatory candidates. We first restricted our search to variants in the 16% of the genome (471 Mb) harbouring molecular evidence of regulatory functions or evolutionary conservation in any of the resources leveraged by FINSURF (Table B in S1 text). This filter assumes that the remaining 84% of the genome which have never been associated with regulatory evidence are unlikely to be functional, and greatly speeds up computation. All pathogenic variants were retained, and they remain concentrated in the top ranking variants of this subset, with 74% in the top 5% (Mann-Whitney test, p value = 2.27 10^−23^), but still among a vast excess of false positives. Then, we relied on the OMIM database [31] to establish lists of potentially deregulated genes for each of the 30 diseases, and only retained variants predicted by FINSURF to interact with those genes. For example, cerebral amyloid angiopathy is caused by the β-amyloid precursor protein (APP) [43], whose gene is predicted to interact with 428 candidate variants in this realistic setting. FINSURF correctly ranks the pathogenic variant in first position (Fig 4B). Overall, across the 30 diseases included in the study, between 3 and 495 variants from the synthetic genome are predicted to interact with known disease genes (average: 115). FINSURF ranks the causative pathogenic variants in first position in 11 instances. For 8 other diseases, FINSURF ranked the causative mutation in the top 5 variants, and in 4 additional cases, the causative mutation was in the top 10 variants. In summary, FINSURF ranked the pathogenic variant(s) within the first ten candidates for 22 out of 30 disease cases, making it the first non-coding variant predictor performing accurately in a practical, realistic setting. Of note, many of the correctly identified pathogenic variants have comparable scores with FINSURF (Fig 4B) and other methods (panels b-d of Fig E in S1 Text), highlighting how target gene predictions built into FINSURF are a major contributor to identifying relevant non-coding mutations in disease contexts.

In two disease cases, FINSURF failed to identify the pathogenic mutation because the variants are not predicted to interact with disease gene(s). Of these, the mutation linked to Pierre Robin Sequence is highly scored by FINSURF (0.808) but is predicted to interact with KCNJ2 and KCNJ16 instead of *SOX9*, the documented causal gene. Interestingly, this variant is located 1.4 Mb away from *SOX9* [44], and its functional involvement in Pierre Robin Sequence may be conditional as this variant is also present in non-affected controls [45]. Further, the two regulatory mutations associated to Maturity Onset Diabetes of the Young type 11 (MODY11) have only been shown to reduce the expression of a reporter gene *in vitro* and have no formally demonstrated role in the pathology [46]. Together, these results suggest that our methodology integrating regulatory features and target predictions shows high discrimination and accuracy to identify relevant non-coding disease variants.

## Discussion

We developed FINSURF to score variants in the human genome in the context of medical genetics, delivering an efficient strategy to identify regulatory non-coding mutations likely to cause a diagnosed disease. While a growing number of machine-learning models have been developed in recent years to identify functional non-coding variants [47,48], few if any are applicable in practical clinical contexts, because of their design and control choices. A classical problem when designing an experiment, choices of positive and negative controls are not only paramount for model accuracy and precision, but they must also be tailored to the question that the model aims to solve. We know that a typical WGS dataset contains thousands of benign variants that occur in conserved or epigenetically active regions with characteristics of regulatory sequences [8]. Our goal is to identify a unique mutation among those that likely causes a disease. While developing FINSURF, we paid a high degree of attention to the design of a negative control dataset, and its effects on model performances and generalization. Positive controls, e.g. validated non-coding regulatory variants, remain few and far between in the literature. As a result, most models including FINSURF are trained on similarly limited datasets. However, we show here that the non-coding regulatory variants in the HGMD database are quite heterogeneous in terms of genomic location and biological features (Fig 3), suggesting that FINSURF can capture a varied range of functional variants. Possibly less appreciated, negative controls are also crucial to developing a successful model. Randomly chosen benign human variants, or solely based on population frequency, result in models that successfully discriminate negatives from positives but lack precision within broad regulatory regions. On the other hand, benign variants closely matched to positive controls achieve excellent local discrimination but result in overly specific models. We note that previous models have been trained on sometimes elaborately sampled negative variants [25,49], but the theoretical justifications and the consequences of those choices on model performance have rarely been explored. These considerations have been eclipsed by over-reliance on ROC curves to assess performance, but ought to be properly addressed. FINSURF was explicitly tested both at the general and the local level using different sets of controls to ensure high performances genome-wide yet serve the purpose of identifying pathogenic mutations from a background of variants in similar genomic contexts.

Nonetheless, the concept of regulatory sequence covers multiple situations. From developmental enhancers with strong effects on gene expression and organism fitness to redundant shadow enhancers, ultimately the regulatory potential of a genomic sequence is likely to be a continuous property rather than a binary characteristic. This is consistent with the distribution of FINSURF scores observed during performance tests (Fig 2) where 6.4% of benign variants obtain scores above the optimal separation threshold of 0.51, while 41.4% of pathogenic mutations are below this threshold and therefore not distinguishable from benign variants. Solely relying on a score and threshold to identify relevant regulatory mutations from whole-genome sequences is probably bound to fail in the vast majority of medical genomics applications. To alleviate this issue, we provide an efficient strategy to first prioritize context-relevant candidate variants, and then characterise the individual functional profiles of each candidate identified by the model for further interpretation. Combining information from FINSURF scores, target predictions and disease aetiology, we are able to correctly identify the causative regulatory mutation as a top candidate in an array of 22 pathologies representative of the state-of-the-art on non-coding pathogenic mutations.

This strategy allows users to integrate the discovery power of machine learning models with prior knowledge on gene-to-phenotype associations to restrict and refine searches for candidate genetic variants. Although FINSURF can be used as an agnostic approach to explore regulatory variants associated to any gene, we show that predicted target genes aggregated from molecular and evolutionary evidence by FINSURF can be decisive in identifying variants that interact with genes of interest. This procedure effectively makes it possible to leverage the vast knowledge accumulated from gene functional exploration, knock-outs and perturbation experiments in human and animal models, which can be extracted from the literature or from dedicated databases [50], and extends it to the more obscure non-coding fraction of the genome. While current sets of such mutations arguably suffer from ascertainment bias, FINSURF may significantly broaden the pool of relevant candidates for future clinical analyses compared to prevailing approaches where exon-proximal variants are prioritized.

### Web resources

FINSURF is available as an online application at https://www.finsurf.bio.ens.psl.eu/ where users can load their variants in VCF format as well as optional lists of relevant target genes. The webserver returns an interactive list of ranked candidate non-coding variants, and allows users to investigate their functional characteristics through Feature Importance graphs and links to custom tracks in the UCSC web browser.

## Supporting information

S1 Text Supporting Information include 3 tables and 6 figures. **Table A.** Sources of functional annotations used by FINSURF. **Table B.** Sources of regulatory regions associated to putative target genes used by FINSURF. **Table C**. Variant scoring methods compared to FINSURF. **Fig A.** Design and cross-performance evaluation of the random forests models trained with the three approaches for sampling negative control variants. As described in the methods, the Random sampling does not correct for the differences in proportions between positive and negative controls in the different genomic annotations, while the Adjusted model corrects for this. The cross-performance of the models is evaluated by comparison of the ROC and precision-recall curves, recalculated from the 10 fold cross validation step of each model (which correspond to the pairs of curves on the diagonal). A given column corresponds to the application of a model on the 10-fold subsets of variants obtained under the different samples methods (with filtering of overlapping variants between training and validation subsets, as described in the Material and Methods). Note that to allow the comparison of performances, a second undersampling of negative control variants was performed in the validation subset for the Random and Adjusted models, in order to match the observed proportion of 12.5% of positive controls in the Local sampling dataset. The area under the curves of the average curves is reported in the two tables for each combination of model and validation dataset. **Fig B**. Comparison of the cross-validation scheme for separating training and validation sets of variants. Separability plots include the Receiver Operating Curve (ROC) and Precision Recall Curve (PRC) averaged over 10-fold cross-validation, as well as the density plot of positive and negative controls over the range of FINSURF predicted scores. a. Results from a 10-fold cross-validation where variants were separated taking into account their localisation within cytogenetic bands. Variants (whether negative or positive controls) within a certain cytogenetic band are randomly assigned to one of the 10 folds for the cross-validation, ensuring that information from their local genomic context will not leak from the training set to the validation set. This approach is the one retained for all cross-validations experiments in our analyses. Note that these ROC and PRC are the one presented in the Figs A and B in S1 Text. b. Results from a 10-fold “Stratified” cross-validation, where only the imbalance of positive and negative variants was taken care of by the stratification procedure. The much better performance and cleaner separation between classes highlights data leakage from separating at random into train and validation sets variants that might share common genomic context. **Fig C.** Comparison between FINSURF (red lines) and eight other methods also designed to functionally interpret non-coding regulatory variants. a. Comparison of performances on the training dataset of FINSURF “Adjusted”. The subsets of variants generated during the 10-fold cross-validation were re-used, by scoring the positive and negative control variants with the other methods. At no time was FINSURF tested on variants used during training. Variants for which a score could not be computed by any one method (e.g. lack of required annotations) were discarded for all methods. The average performance curve of the 10-folds are reported. We note the high precision of the ReMM Genomiser model, which can be explained by the overlap between the training variants of this model and the HGMD variants used by FINSURF. b. Comparison of performances on the subset of 92 Genomiser variants, of which 30 are discarded due to missing scores by at least one method. Negative control variants were re-sampled following the Adjusted sampling procedure, and those found in the training dataset of the FINSURF model were removed. Note that the ROC curve is the one presented in the Fig 2d. **Fig D.** Feature importance of the 41 features in the FINSURF Adjusted model. Features are listed on the x-axis and grouped in four categories: green for nucleotide sequence features, purple for evolutionary sequence conservation features, red for functional genomics features and blue for putative enhancer–promoter association features. **Fig E**. Analysis of the 878 positive control variants of the FINSURF model through their feature contribution profiles. Contributions to the prediction score were calculated for each variant, and k-means clustering was applied. a. Different number of clusters were explored, and the finale number K = 7 was selected from the joint minimization of the inertia and maximization of the silhouette. b. Distribution of scores of variants per cluster, colored by the predicted class when applying the optimal threshold of 0.51. c. Average functional profile of the 7 clusters. These profiles highlight how feature contributions relate to the raw values of the features, as processed by the random forests. For each cluster, the raw values were normalized, and averaged from the variants within the cluster (colored bars). Reference values (dark grey bars) correspond to the average from the rest of the training dataset (i.e. positive controls in other clusters and negative controls). The color of the bars is based on the average contributions of the features in each cluster. **Fig F**. Predicting highly pathogenic disease variant in genomic context. a. Box-plots representation and individual FINSURF score values for 92 known pathogenic variants from the Genomiser dataset but absent from the FINSURF training set. While 43 variants are very close (≤ 1kb) to one of the latter (left), the remaining 49 are sufficiently far (> 1kb) to be considered to overlap independent annotations. Of these a subset of 38 variants are also predicted to reside in a regulatory element targeting a gene that is not in the set of targets of variants in the FINSURF training set (“not seen”). Since the two subsets (N = 11 and N = 38) show largely overlapping score distributions, they were all used for classification performance in a genomic context. b. Classification performance comparisons between FINSURF and eight other methods on the set of 49 positive variants described in (a): left, ROC; middle, PRC; right, scatterplot of ROC versus PRC AUC values for graphs shown on the left and middle panels. We dropped the 3 variants from the MODY11 and PRS diseases, as they were located in regulatory regions not targeting the reported disease gene. Note that only 37 variants were eventually scored by all 9 methods and could be used for the analysis. The negative set used here were the 875 Platinum variants also residing in the same predicted regulatory regions as the 37 positives. c. Table reporting the number of OMIM diseases where at least one of the Genomiser variants is found in the top 10 candidates, after the native score from each method was filtered through Enhancer-Gene interactions that are part of the FINSURF approach. The first row indicates these counts for each method, where Genomiser variants are ranked among Platinum variants from within regulatory regions targeting the disease genes from each disease. The total of 30 OMIM disease is reduced for some methods, as ranking is impossible if they do not provide scores for all Genomiser variants. The second row shows these counts for the subset of OMIM diseases where all variants are scored by all methods. Note that here 59% of Platinum variants are discarded, reducing the space search in an unrealistic fashion. d. Comparison of ranks for the 37 positive variants among the 875 negatives, based on scores attributed by FINSURF and eight other methods, after the native score from each method was filtered through Enhancer-Gene interactions that are part of the FINSURF approach. Ranks were normalised between 0 and 100 for each method (y-axis). Colours represent different diseases. The graph shows that there is little correlation between variant ranks among the different methods.
(DOCX)
