# Supplementary material for: Classification of non-coding variants with high pathogenic impact
Source: PLoS Genet. 2022 Apr 29;18(4):e1010191. doi: 10.1371/journal.pgen.1010191 (PMC9094564; doi:10.1371/journal.pgen.1010191)
Supplement: S1 Text — Table A. Sources of functional annotations used by FINSURF. Table B. Sources of regulatory regions associated to putative target genes used by FINSURF. Table C. Variant scoring methods compared to FINSURF. Fig A. Design and cross-performance evaluation of the random forests models trained with the three approaches for sampling negative control variants. As described in the methods, the Random sampling does not correct for the differences in proportions between positive and negative controls in the different genomic annotations, while the Adjusted model corrects for this. The cross-performance of the models is evaluated by comparison of the ROC and precision-recall curves, recalculated from the 10 fold cross validation step of each model (which correspond to the pairs of curves on the diagonal). A given column corresponds to the application of a model on the 10-fold subsets of variants obtained under the different samples methods (with filtering of overlapping variants between training and validation subsets, as described in the Material and Methods). Note that to allow the comparison of performances, a second undersampling of negative control variants was performed in the validation subset for the Random and Adjusted models, in order to match the observed proportion of 12.5% of positive controls in the Local sampling dataset. The area under the curves of the average curves is reported in the two tables for each combination of model and validation dataset. Fig B. Comparison of the cross-validation scheme for separating training and validation sets of variants. Separability plots include the Receiver Operating Curve (ROC) and Precision Recall Curve (PRC) averaged over 10-fold cross-validation, as well as the density plot of positive and negative controls over the range of FINSURF predicted scores. a. Results from a 10-fold cross-validation where variants were separated taking into account their localisation within cytogenetic bands. Variants (whether negative or posit [file pgen.1010191.s001.docx]

**Classification of non-coding variants with high pathogenic impact**

Lambert Moyon^1^, Camille Berthelot^1^, Alexandra Louis^1^, Nga Thi Thuy Nguyen^1^ and Hugues Roest Crollius^1,*^

^1^ Ecole Normale Supérieure, PSL Research University, CNRS, Inserm, Institut de Biologie de l’Ecole Normale Supérieure (IBENS), Paris, France

* hrc@bio.ens.psl.eu

**Supporting Information**

**Design of the three models for negative control sampling**

When training machine learning frameworks, imbalance and unequal distributions in the training data are major issues, which have been minimally addressed in the past. The set size and genomic distribution of positive controls is unfortunately limited by data availability in the current state-of-the-art. A potential consequence is that models may largely learn to discriminate based on distance from the nearest gene, which is not particularly useful nor does it require machine learning to achieve.

The Random model confronts random non-functional variants to the positive controls, and evaluates the baseline: a good model should not be specific to any subcategory of genomic regions, and should work genome-wide. At each training iteration, 880 negative and 880 positive controls are sampled randomly with replacement in a balanced set design. As there are 1,000 training iterations and the negative pool is very large (38 million), we estimate that ~880,000 negative variants are sampled in the training, with minimal resampling.

The Local model confronts the positive variants to closely-located non-functional variants, to ensure that the model does not simply discriminate based on genomic location (especially relative to gene distance), but is able to distinguish true functional variants from their immediate surroundings. Indeed, it is not expected that every base pair in regulatory elements has functional consequences when mutated. We chose to sample negative controls within 1kb, which is the size of an average non-coding regulatory element as determined from epigenomic information (e.g. histone modifications). We find that such closely-located variants are much harder to discriminate, as expected, because they frequently share functional features spanning more than 1kb.

An evaluation of cross-performance (Fig A) indicated that each model performs poorly on the training dataset of the other. This shows that neither the Random nor the Local models generalize to the complete range of situations encountered in real settings. The Adjusted model however uses a composite set of negative controls (randomly and locally sampled), and achieves high performances on all training sets regardless of the genomic distribution of the variants (Fig A).

| **Feature** | **Description** | **Group** | **Distribution** | **Reference** |
| --- | --- | --- | --- | --- |
| Transition/Transversion | Nature of the variants (0 for transitions, 1 for transversions ; indels are set to 2). | Sequence properties | Discrete | This study |
| CG dinucleotide | CG dinucleotide locations. |  | Binary | This study |
| CpG islands | CpG islands from the UCSC. |  | Binary | [1] |
| PhyloP 100-way | PhyloP score computed from 100 vertebrate genomes’ alignments | Conservation | Continuous | [1,2] |
| PhyloP 20-way | PhyloP score computed from 20 primate genomes’ alignments |  | Continuous | [1,2] |
| PhastCons 100-way | PhastCons score computed from 100 vertebrate genomes’ alignments |  | Continuous | [1,3] |
| PhastCons 20-way | PhastCons score computed from 20 primate genomes’ alignments |  | Continuous | [1,3] |
| CDTS | Context-dependent tolerance score representing the tolerance to genetic variation, evaluated at the population level. |  | Continuous | [4] |
| GERP score | GERP score |  | Continuous | [5] |
| GERP element | Regions identified as conserved element. |  | Binary | [5] |
| DNase-sensitive cluster | Score associated to DNaseI Hypersensitivity Clusters in 125 cell types from ENCODE. | Biochemical evidence | Continuous | [6] |
| Promoter | Number of the considered state at given location across 98 cell types from Roadmap Epigenomics. |  | Discrete | [7] |
| Transcribed |  |  | Discrete | [7] |
| Enhancer |  |  | Discrete | [7] |
| ZNF Genes & repeats |  |  | Discrete | [7] |
| Heterochromatin |  |  | Discrete | [7] |
| Repressed |  |  | Discrete | [7] |
| Quiescent |  |  | Continuous | [7] |
| H3K4me1 fold-change | Median of Fold Change value per position for the considered histone mark across 98 cell types from Roadmap Epigenomics. |  | Continuous | [7] |
| H3K4me3 fold-change |  |  | Continuous | [7] |
| H3K27ac fold-change |  |  | Continuous | [7] |
| TFBS count | From predicted TFBS overlapped by a variant, two information are extracted : the number of predicted TFBS, and the maximum score of site prediction. |  | Discrete | [8] |
| TFBS max score |  |  | Continuous | [8] |
| Conserved TFBS count |  |  | Discrete | UCSC track |
| Conserved TFBS max score |  |  | Continuous | UCSC track |
| Clustered TFBS count |  |  | Discrete | UCSC track |
| Clustered TFBS max score |  |  | Continuous | UCSC track |
| PEGASUS score | From predicted regulatory regions overlapped by a variant, two information are extracted : the score associated to the regulatory region, and the number of targets. When multiple regions from a dataset overlap, the maximum score is retrieved.  GeneHancer provides with an additional score for the predictions of interactions, which is extracted as “max score targets”. | Gene associations | Continuous | [9] |
| PEGASUS target count |  |  | Discrete | [9] |
| FANTOM5 max score |  |  | Continuous | [10] |
| FANTOM5 target count |  |  | Discrete | [10] |
| GeneHancer max score |  |  | Continuous | [11] |
| GeneHancer target count |  |  | Discrete | [11] |
| GeneHancer max score targets |  |  | Continuous | [11] |
| FOCS_FANTOM5 max score |  |  | Continuous | [12] |
| FOCS_FANTOM5 target count |  |  | Discrete | [12] |
| FOCS_GroSeq max score |  |  | Continuous | [12] |
| FOCS_GroSeq target count |  |  | Discrete | [12] |
| FOCS_Roadmap max score |  |  | Continuous | [12] |
| FOCS_Roadmap target count |  |  | Discrete | [12] |
| Ratio shared targets | At positions overlapped by regulatory regions of from multiple source, we divide the number of genes predicted as a target by more than 1 source, by the total number of target genes across sources. |  | Continuous | This study |

**Table A: Sources of functional annotations used by FINSURF**

| **Source** | **Description** | **Number of bases** | **Reference** |
| --- | --- | --- | --- |
| GENCODE v29liftHg19 | Extracted 5’UTR and 3’UTR regions, as well as proximal regulatory regions set as the 2,000bp before the first position of annotated genes (both coding and non-coding). | 128,404,642 | [13] |
| PEGASUS | Predicted regulatory regions identified from conserved non-coding elements. | 68,593,981 | [9] |
| FANTOM5 | Robust enhancer-promoter associations based on correlation of expression levels measured by CAGE. | 8,444,398 | [10] |
| GeneHancer | Unified predicted regulatory regions from a set of 4 sources : Ensembl regulatory built, FANTOM5 permissive enhancers, VISTA enhancers, and ENCODE proximal and distal enhancers. | 312,286,188 | [11] |
| FOCS FANTOM5 | Predictions through the FOCS pipeline of regulatory associations from FANTOM5 expressed regions. | 5,131,199 | [12] |
| FOCS GroSeq | Predictions through the FOCS pipeline of regulatory associations from global run-on sequencing datasets. | 21,636,242 | [12] |
| FOCS Roadmap | Predictions through the FOCS pipeline of regulatory associations from DHS datasets from Roadmap Epigenomics. | 13,401,492 | [12] |

**Table B: Sources of regulatory regions associated to putative target genes used by FINSURF**

| **Name** | **Model** | **Training scheme** | **Evaluation** | **Ref** |
| --- | --- | --- | --- | --- |
| Genomiser | Supervised classification with an ensemble of random forests. | Manual curation of a set of functional variants (note : a large part overlaps with HGMD), against nearly fixed human-specific variants identified within the primate lineage, taken as negative controls. |  | [14] |
| NCBoost | Supervised classification with gradient-boosting of decision trees (XGBoost). |  |  | [15] |
| FATHMM-MKL | Supervised classification with a Support Vector Machine using a Multi-Kernel learning approach. | Positive controls are taken from HGMD. Negative controls are taken from the 1,000 genome project : variants with mAF>1 %, at a maximum distance of 1kb of a positive control. |  | [16] |
| Eigen | Unsupervised learning |  |  | [17] |
| Linsight |  |  |  | [18] |
| FitCons | Evaluation of sequence-constraint at base-level within different genomic regions identified from biochemical features. |  |  | [19] |
| CADD | Supervised classification with a Support Vector Machine using a linear kernel. |  |  | [20] |

**Table C: Variant scoring methods compared to FINSURF**


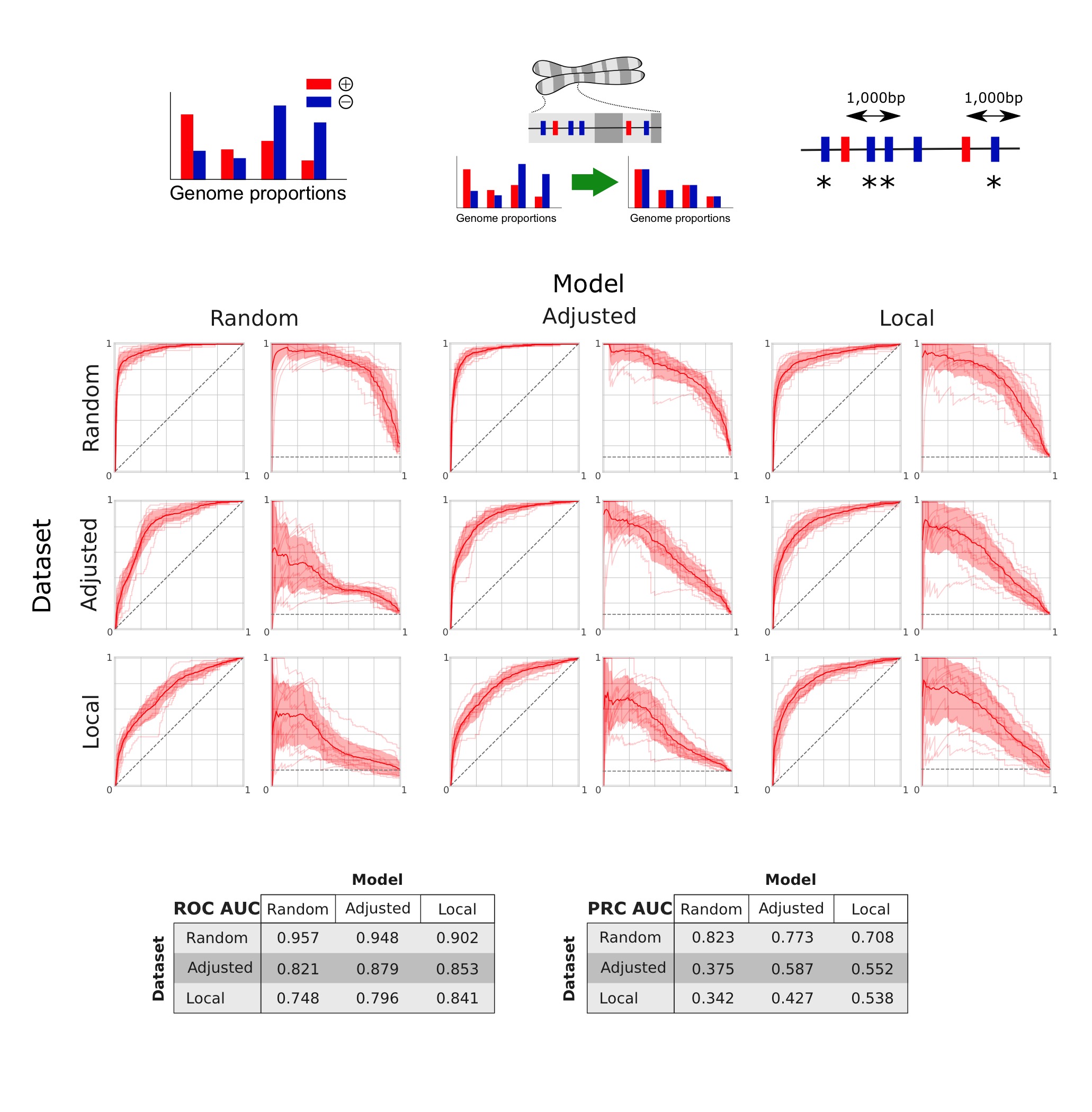


**Fig A.** Design and cross-performance evaluation of the random forests models trained with the three approaches for sampling negative control variants. As described in the methods, the Random sampling does not correct for the differences in proportions between positive and negative controls in the different genomic annotations, while the Adjusted model corrects for this. The cross-performance of the models is evaluated by comparison of the ROC and precision-recall curves, recalculated from the 10 fold cross validation step of each model (which correspond to the pairs of curves on the diagonal). A given column corresponds to the application of a model on the 10-fold subsets of variants obtained under the different samples methods (with filtering of overlapping variants between training and validation subsets, as described in the methods). Note that to allow the comparison of performances, a second undersampling of negative control variants was performed in the validation subset for the Random and Adjusted models, in order to match the observed proportion of 12.5% of positive controls in the Local sampling dataset. The area under the curves of the average curves is reported in the two tables for each combination of model and validation dataset.


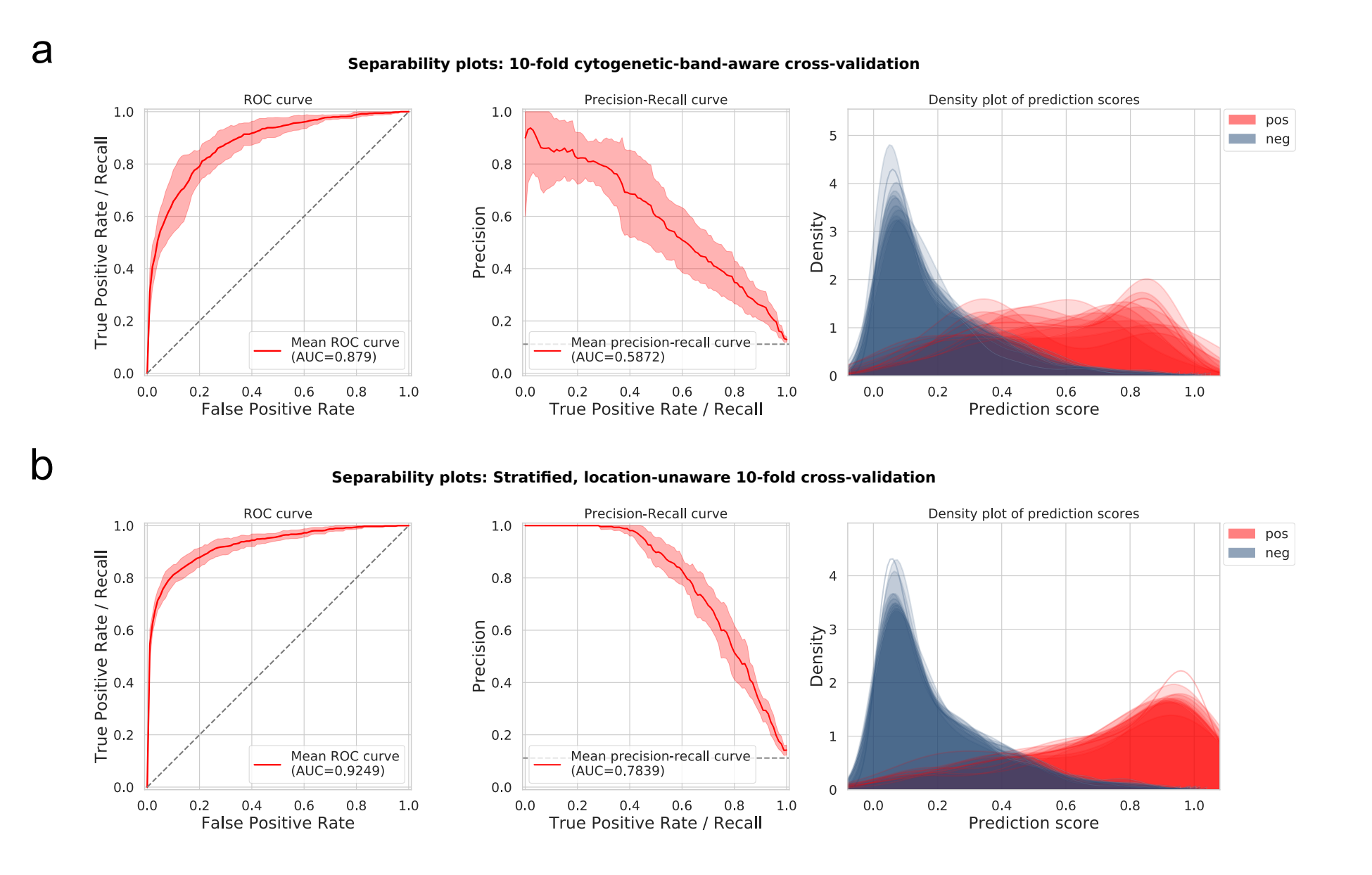
**Fig B.** Comparison of the cross-validation scheme for separating training and validation sets of variants. Separability plots include the Receiver Operating Curve (ROC) and Precision Recall Curve (PRC) averaged over 10-fold cross-validation, as well as the density plot of positive and negative controls over the range of FINSURF predicted scores. **a.** Results from a 10-fold cross-validation where variants were separated taking into account their localisation within cytogenetic bands. Variants (whether negative or positive controls) within a certain cytogenetic band are randomly assigned to one of the 10 folds for the cross-validation, ensuring that information from their local genomic context will not leak from the training set to the validation set. This approach is the one retained for all cross-validations experiments in our analyses. Note that these ROC and PRC are the one presented in the Fig A in S1 Text and Fig 2. **b.** Results from a 10-fold “Stratified” cross-validation, where only the imbalance of positive and negative variants was taken care of by the stratification procedure. The much better performance and cleaner separation between classes highlights data leakage from separating at random into train and validation sets variants that might share common genomic context.

**Fig C.** Comparison between FINSURF (red lines) and eight other methods also designed to functionally interpret non-coding regulatory variants. **a**. Comparison of performances on the training dataset of FINSURF “Adjusted”. The subsets of variants generated during the 10-fold cross-validation were re-used, by scoring the positive and negative control variants with the other methods. At no time was FINSURF tested on variants used during training. Variants for which a score could not be computed by any one method (e.g. lack of required annotations) were discarded for all methods. The average performance curve of the 10-folds are reported. We note the high precision of the ReMM Genomiser model, which can be explained by the overlap between the training variants of this model and the HGMD variants used by FINSURF. **b**. Comparison of performances on the subset of 92 Genomiser variants, of which 30 are discarded due to missing scores by at least one method. Negative control variants were re-sampled following the Adjusted sampling procedure, and those found in the training dataset of the FINSURF model were removed. Note that the ROC curve is the one presented in the Fig 2d.

**Fig D.** Feature importance of the 41 features in the FINSURF Adjusted model. Features are listed on the x-axis and grouped in four categories: green for nucleotide sequence features, purple for evolutionary sequence conservation features, red for functional genomics features and blue for putative enhancer – promoter association features.


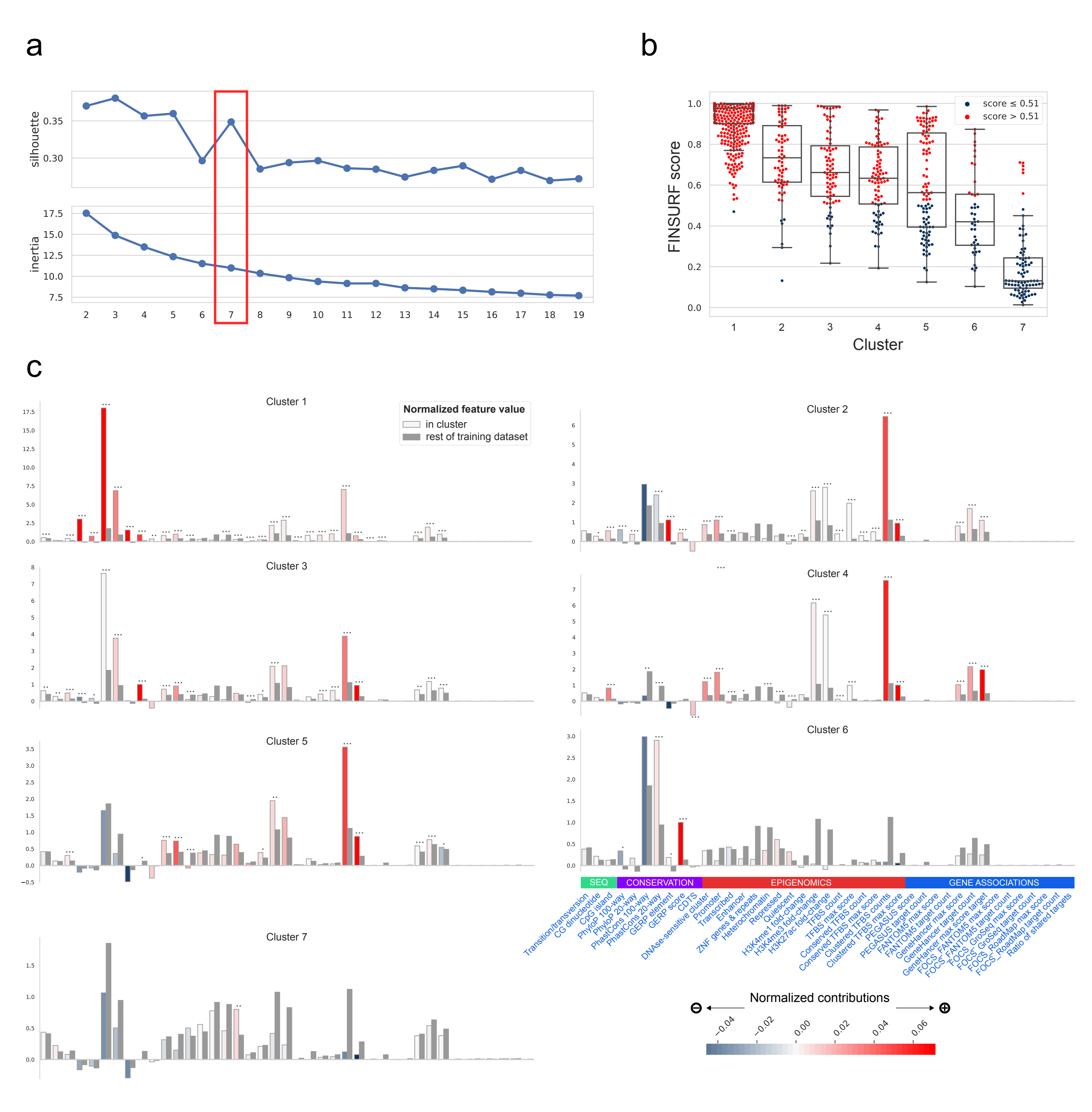


**Fig E**. Analysis of the 878 positive control variants of the FINSURF model through their feature contribution profiles. Contributions to the prediction score were calculated for each variant, and k-means clustering was applied. **a**. Different number of clusters were explored, and the finale number K=7 was selected from the joint minimization of the inertia and maximization of the silhouette. **b**. Distribution of scores of variants per cluster, colored by the predicted class when applying the optimal threshold of 0.51. **c**. Average functional profile of the 7 clusters. These profiles highlight how feature contributions relate to the raw values of the features, as processed by the random forests. For each cluster, the raw values were normalized, and averaged from the variants within the cluster (colored bars). Reference values (dark grey bars) correspond to the average from the rest of the training dataset (i.e. positive controls in other clusters and negative controls). The color of the bars is based on the average contributions of the features in each cluster.


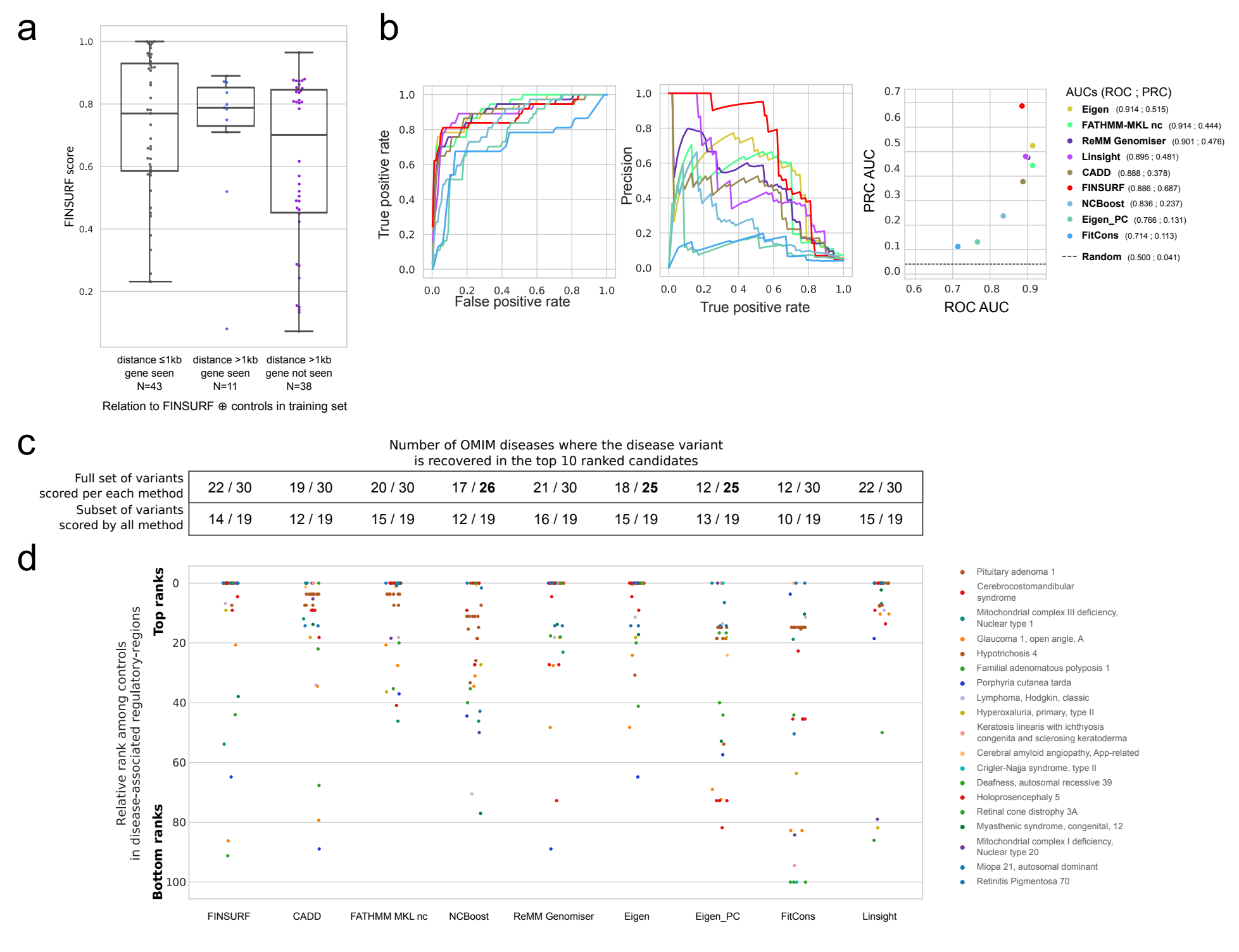


**Fig F.** Predicting highly pathogenic disease variant in genomic context. **a**. Box-plots representation and individual FINSURF score values for 92 known pathogenic variants from the Genomiser dataset but absent from the FINSURF training set. While 43 variants are very close (≤ 1kb) to one of the latter (left), the remaining 49 are sufficiently far (> 1kb) to be considered to overlap independent annotations. Of these a subset of 38 variants are also predicted to reside in a regulatory element targeting a gene that is not in the set of targets of variants in the FINSURF training set (“not seen”). Since the two subsets (N=11 and N=38) show largely overlapping score distributions, they were all used for classification performance in a genomic context. **b**. Classification performance comparisons between FINSURF and eight other methods on the set of 49 positive variants described in (a): left, ROC; middle, PRC; right, scatterplot of ROC versus PRC AUC values for graphs shown on the left and middle panels. We dropped the 3 variants from the MODY11 and PRS diseases, as they were located in regulatory regions not targeting the reported disease gene. Note that only 37 variants were eventually scored by all 9 methods and could be used for the analysis. The negative set used here were the 875 Platinum variants also residing in the same predicted regulatory regions as the 37 positives. **c.** Table reporting the number of OMIM diseases where at least one of the Genomiser variants is found in the top 10 candidates, after the native score from each method was filtered through Enhancer-Gene interactions that are part of the FINSURF approach. The first row indicates these counts for each method, where Genomiser variants are ranked among Platinum variants from within regulatory regions targeting the disease genes from each disease. The total of 30 OMIM disease is reduced for some methods, as ranking is impossible if they do not provide scores for all Genomiser variants. The second row shows these counts for the subset of OMIM diseases where all variants are scored by all methods. Note that here 59% of Platinum variants are discarded, reducing the space search in an unrealistic fashion. **d**. Comparison of ranks for the 37 positive variants among the 875 negatives, based on scores attributed by FINSURF and eight other methods, after the native score from each method was filtered through Enhancer-Gene interactions that are part of the FINSURF approach. Ranks were normalised between 0 and 100 for each method (y-axis). Colours represent different diseases. The graph shows that there is little correlation between variant ranks among the different methods.

**References**

1. Lee CM, Barber GP, Casper J, Clawson H, Diekhans M, Gonzalez JN, et al. UCSC Genome Browser enters 20th year. Nucleic Acids Res. 2020;48: D756–D761. doi:10.1093/nar/gkz1012

2. Pollard KS, Hubisz MJ, Rosenbloom KR, Siepel A. Detection of nonneutral substitution rates on mammalian phylogenies. Genome Res. 2010;20: 110–121. doi:10.1101/gr.097857.109

3. Siepel A, Bejerano G, Pedersen JS, Hinrichs AS, Hou M, Rosenbloom K, et al. Evolutionarily conserved elements in vertebrate, insect, worm, and yeast genomes. Genome Res. 2005;15: 1034–1050. doi:10.1101/gr.3715005

4. di Iulio J, Bartha I, Wong EHM, Yu HC, Lavrenko V, Yang D, et al. The human noncoding genome defined by genetic diversity. Nature genetics. 2018. doi:10.1038/s41588-018-0062-7

5. Cooper GM, Stone EA, Asimenos G, Green ED, Batzoglou S, Sidow A. Distribution and intensity of constraint in mammalian genomic sequence. Genome Res. 2005;15: 901–913. doi:10.1101/gr.3577405

6. Thurman RE, Rynes E, Humbert R, Vierstra J, Maurano MT, Haugen E, et al. The accessible chromatin landscape of the human genome. Nature. 2012;489: 75–82. doi:10.1038/nature11232

7. Kundaje A, Meuleman W, Ernst J, Bilenky M, Yen A, Heravi-Moussavi A, et al. Integrative analysis of 111 reference human epigenomes. Nature. 2015;518: 317–330. doi:10.1038/nature14248

8. Zerbino DR, Wilder SP, Johnson N, Juettemann T, Flicek PR. The ensembl regulatory build. Genome Biology. 2015;16: 56. doi:10.1186/s13059-015-0621-5

9. Clément Y, Torbey P, Gilardi-Hebenstreit P, Crollius HR. Enhancer-gene maps in the human and zebrafish genomes using evolutionary linkage conservation. Nucleic Acids Res. 2020;48: 2357–2371. doi:10.1093/nar/gkz1199

10. Andersson R, Gebhard C, Miguel-Escalada I, Hoof I, Bornholdt J, Boyd M, et al. An atlas of active enhancers across human cell types and tissues. Nature. 2014;507: 455–461. doi:10.1038/nature12787

11. Fishilevich S, Nudel R, Rappaport N, Hadar R, Plaschkes I, Iny Stein T, et al. GeneHancer: genome-wide integration of enhancers and target genes in GeneCards. Database (Oxford). 2017;2017. doi:10.1093/database/bax028

12. Hait TA, Amar D, Shamir R, Elkon R. FOCS: a novel method for analyzing enhancer and gene activity patterns infers an extensive enhancer-promoter map. Genome Biology. 2018;19: 56. doi:10.1186/s13059-018-1432-2

13. Harrow J, Frankish A, Gonzalez JM, Tapanari E, Diekhans M, Kokocinski F, et al. GENCODE: The reference human genome annotation for The ENCODE Project. Genome Research. 2012;22: 1760–1774. doi:10.1101/gr.135350.111

14. Smedley D, Schubach M, Jacobsen JOB, Köhler S, Zemojtel T, Spielmann M, et al. A Whole-Genome Analysis Framework for Effective Identification of Pathogenic Regulatory Variants in Mendelian Disease. Am J Hum Genet. 2016;99: 595–606. doi:10.1016/j.ajhg.2016.07.005

15. Caron B, Luo Y, Rausell A. NCBoost classifies pathogenic non-coding variants in Mendelian diseases through supervised learning on purifying selection signals in humans. Genome Biol. 2019;20: 32. doi:10.1186/s13059-019-1634-2

16. Rogers MF, Shihab HA, Mort M, Cooper DN, Gaunt TR, Campbell C. FATHMM-XF: accurate prediction of pathogenic point mutations via extended features. Bioinformatics. 2018;34: 511–513. doi:10.1093/bioinformatics/btx536

17. Ionita-Laza I, McCallum K, Xu B, Buxbaum JD. A spectral approach integrating functional genomic annotations for coding and noncoding variants. Nature Genetics. 2016;48: 214–220. doi:10.1038/ng.3477

18. Huang Y-F, Gulko B, Siepel A. Fast, scalable prediction of deleterious noncoding variants from functional and population genomic data. Nat Genet. 2017;49: 618–624. doi:10.1038/ng.3810

19. Gulko B, Hubisz MJ, Gronau I, Siepel A. A method for calculating probabilities of fitness consequences for point mutations across the human genome. Nat Genet. 2015;47: 276–283. doi:10.1038/ng.3196

20. Kircher M, Witten DM, Jain P, O’Roak BJ, Cooper GM, Shendure J. A general framework for estimating the relative pathogenicity of human genetic variants. Nature Genetics. 2014;46: 310–315. doi:10.1038/ng.2892
